# Emerging clinical evidence of a dual role for Ang-2 and VEGF-A blockade with faricimab in retinal diseases

**DOI:** 10.1007/s00417-024-06695-4

**Published:** 2024-12-21

**Authors:** Varun Chaudhary, Florie Mar, Manuel J. Amador, Andrew Chang, Kara Gibson, Antonia M. Joussen, Judy E. Kim, Junyeop Lee, Philippe Margaron, Insaf Saffar, David Wong, Charles Wykoff, Srinivas Sadda

**Affiliations:** 1 Department of Surgery, McMaster University, Hamilton, ON Canada; 2 Genentech, Inc, South San Francisco, CA USA; 3 Sydney Retina Clinic, Sydney Eye Hospital, University of Sydney, University of NSW, Sydney, Australia; 4 Roche Products Ltd, Welwyn Garden City, UK; 5 Department of Ophthalmology, Charité – Universitätsmedizin Berlin, Berlin, Germany; 6 University of Texas Southwestern Medical Center, Dallas, TX USA; 7 Asan Medical Center, University of Ulsan, College of Medicine, Seoul, South Korea; 8 F. Hoffmann-La Roche Ltd, Basel, Switzerland; 9 Department of Ophthalmology and Vision Sciences, Temerty Faculty of Medicine, University of Toronto, Toronto, ON Canada; 10 Retina Consultants of Texas, Retina Consultants of America, Blanton Eye Institute, Methodist Hospital, Houston, TX USA; 11 Doheny Eye Institute, University of California, Los Angeles, 150 N. Orange Grove Blvd, Suite 232, Pasadena, CA USA

**Keywords:** Ang-2, Diabetic macular edema, Diabetic retinopathy, Neovascular age-related macular degeneration, Retinal vein occlusion, VEGF-A

## Abstract

Anti-vascular endothelial growth factor (VEGF) therapies have transformed the treatment of retinal diseases. However, VEGF signaling is only one component of the complex, multifactorial pathophysiology of retinal diseases, and many patients have residual disease activity despite ongoing anti-VEGF treatment. The angiopoietin/tyrosine kinase with immunoglobulin and epidermal growth factor receptor-2 (Ang/Tie2) signaling pathway is critical to endothelial cell homeostasis, survival, integrity, and vascular stability. Ang-2 can interfere with Ang-1/Tie2 signaling and is increased in several retinal diseases. Lack of Tie2 signaling due to elevated Ang-2 levels drives vascular instability through pericyte dropout, neovascularization, vascular leakage, inflammation, and fibrosis. Although Ang-2 and VEGF can synergistically promote vascular instability and neovascularization, Ang-2 may also mediate vascular instability independently of VEGF. Faricimab is a bispecific antibody designed for intraocular use that inhibits two distinct pathways via Ang-2 and VEGF-A blockade. Clinical biomarkers of vascular instability are important for evaluating disease control and subsequent treatment decisions. These biomarkers include measurement/evaluation with optical coherence tomography (OCT) of intraretinal fluid, subretinal fluid, central subfield thickness, and pigment epithelial detachments (PEDs), and fluorescein angiography imaging of macular leakage and PEDs. Hyperreflective foci (HRF), thought to be representative of activated microglia, indicating an inflammatory microenvironment, and epiretinal membranes (ERMs), a marker for retinal fibrotic proliferation in diabetic macular edema (DME), are both also identified using OCT. Here we summarize data (secondary endpoint and prespecified exploratory analyses as well as post hoc analyses) from six Phase III trials suggest that dual therapy Ang-2/VEGF-A inhibition with faricimab (6 mg) has a greater effect on reducing/resolving biomarkers of vascular instability than aflibercept (2 mg), by both controlling neovascularization and vascular leakage (with resultant resolution of exudation associated with DME, neovascular age-related macular degeneration, and retinal vein occlusion), as well as by targeting inflammation (reduction of HRF in DME) and retinal fibrotic proliferation (reducing the risk of ERMs in eyes with DME). Modulation of both the Ang-2 and VEGF-A pathways with faricimab may therefore provide greater disease control than anti-VEGF monotherapy, potentially leading to extended treatment durability and improved long-term outcomes.

## Abstract (highlights)

**Key messages:**

***What is known*:**

- VEGF signaling is only one component of the multifactorial pathophysiology of retinal diseases; angiopoietin-2 (Ang-2) levels are also elevated, and Ang-2 acts synergistically with or independently of VEGF to mediate vascular instability.
- Faricimab (dual Ang-2/VEGF-A bispecific antibody) showed non-inferior visual gains vs. aflibercept 2 mg (anti-VEGF) with extended durability in six Phase III trials (in diabetic macular edema, neovascular age-related macular degeneration, and retinal vein occlusion) that all met their primary endpoints.

***What is new*:**

- Subsequent secondary, exploratory or post hoc analyses suggest faricimab has a greater effect on reducing/resolving biomarkers of vascular leakage, inflammation, and fibrosis than aflibercept.
- Modulating both the Ang-2 and VEGF-A pathways with faricimab provides greater disease control than anti-VEGF monotherapy alone, potentially leading to extended treatment durability and improved long-term outcomes.

## Multifactorial pathophysiology of retinal disease: evidence from biology

**Fig. 1:**
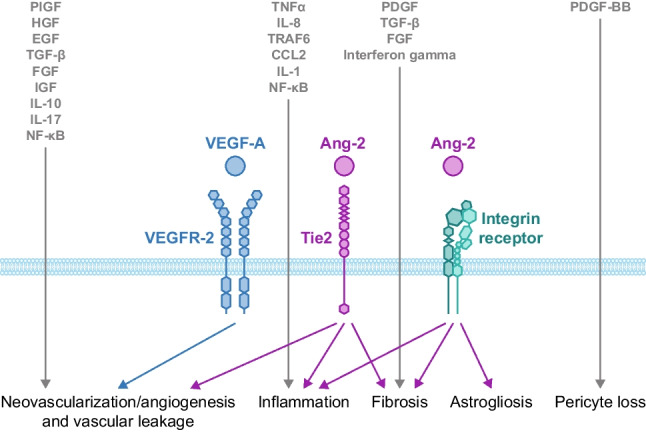
Summary of potential factors involved in retinal diseases [2–4, 7, 9–12]. The roles of Ang-2 and VEGF-A are described in this manuscript. The complexity of retinal disease pathogenesis is further highlighted by the role of numerous other signaling pathways (indicated in grey). Ang-2, angiopoietin-2; CCL2, C–C motif ligand 2; EGF, epidermal growth factor; FGF, fibroblast growth factor; HGF, hepatocyte growth factor; IGF, insulin-like growth factor; IL, interleukin; PDGF, platelet-derived growth factor; PIGF, placental growth factor; TGF, transforming growth factor; TNF, tumor necrosis factor; TRAF6, tumor necrosis factor receptor-associated factor 6; VEGF-A, vascular endothelial growth factor-A

Anti-vascular endothelial growth factor (VEGF) therapies have revolutionized retinal disease treatment; however, VEGF signaling is only one component in the complex pathophysiology of retinal diseases (Fig. 1) [1–3]. VEGF signaling promotes angiogenesis and is modulated by multiple mechanisms. Under healthy, physiologic conditions, the vasculature is generally quiescent except during wound healing and the reproductive cycle [4]. Under pathologic conditions, elevated VEGF levels can occur in response to hypoxia, growth factors, and inflammatory cytokines [3, 5]. This results in neovascularization as well as vascular leakage in neovascular age-related macular degeneration (nAMD), diabetic retinopathy (DR), diabetic macular edema (DME), and retinal vein occlusion (RVO) [6, 7]. VEGF upregulation in nAMD promotes macular neovascularization; the fragility and permeability of these new vessels can result in retinal pigment epithelium detachment (PED), sub-retinal and intra-retinal edema, hemorrhage, and fibrosis [3, 6, 8].

Despite anti-VEGF therapies, a substantial proportion of patients have residual disease activity following ongoing treatment, potentially due to the activity of other pathways and disease mechanisms. For example, the HAWK/HARRIER clinical trial data have shown that in patients with nAMD treated with brolucizumab or aflibercept, 24–39% of patients, respectively, had retinal fluid at 2 years [13]. In VIEW1/VIEW2, only half of patients treated with aflibercept or ranibizumab for nAMD were fluid-free at 96 weeks [14]. Similarly, data of patients with DME treated with aflibercept, bevacizumab or ranibizumab showed that 44–68% had persistent DME at 2 years [15]. Therefore, targeting additional pathways is of important clinical relevance.

The angiopoietin-1/tyrosine kinase with immunoglobulin and epidermal growth factor receptor-2 (Ang-1/Tie2) signaling pathway is critical to endothelial cell (EC) homeostasis, survival, integrity, and vascular stability [3, 7, 16]. Ang-2 can interfere with Ang-1/Tie2 signaling, with increased levels in nAMD, DR, proliferative diabetic retinopathy (PDR), and RVO [17]. Preclinical data have revealed elevated Ang-2 levels in response to stimuli including tumor necrosis factor, VEGF, fibroblast growth factor, shear stress, hyperglycemia, and hypoxia [9, 16, 18, 19].

Elevated Ang-2 levels drives vascular instability through pericyte dropout, neovascularization, and vascular leakage. This leads to inflammation and fibrosis [3, 10, 20, 21].

In mouse models, Ang-2 triggered blood–retinal barrier breakdown in a positive feedback loop while Ang-2 blockade prevented vascular destabilization [22]. Ang-2 can also potentiate VEGF effects by destabilizing vessels (rendering them more vulnerable to VEGF) and facilitating VEGF-mediated EC–EC junction destabilization, further driving neovascularization and vascular leakage [1, 9, 23]. Ang-2 regulates proinflammatory responses and can potentiate the effects of inflammatory cytokines [1, 20, 24]. Ang-2 signaling induces expression of intercellular adhesion molecule-1 and vascular cell adhesion molecule-1, promoting migration and adhesion of leucocytes into inflamed tissues [3]. This in turn results in the release of more inflammatory cytokines, growth cytokines, and vascular permeability factors, leading to altered EC junctions and a compromised blood–retinal barrier [25].

Subretinal fibrosis can be driven by several factors including inflammation, vascular leakage, neovascularization, and hemorrhage, which eventually lead to fibrovascular tissue formation and significant vision loss [26, 27]. Thus, modulating fibrosis development should be an important end goal for treatments that aim to optimize visual outcomes. Elevated Ang-2 levels in vitreous samples from patients with PDR have been shown to correlate with the degree of fibrosis as well as the presence of fibrovascular membranes [10]. Epiretinal membranes (ERMs) are fibrocellular proliferations on the internal limiting membrane of the macula likely caused by glial cell proliferation [28, 29]. ERMs can be either idiopathic, with cell proliferation occurring after posterior vitreous detachment [28], or secondary, as a result of existing retinal diseases such as DR, PDR, proliferative vitreoretinopathy, posterior uveitis, RVO as well as in the context of retinal breaks, retinal detachment surgery and inflammation [28, 29]. ERMs are a marker of retinal fibrotic proliferation in DME [30] and can result in anatomic disruption of the macula and vision loss [29]. Elevated Ang-2 levels have been identified in excised ERMs from eyes with ischemic retinal diseases [31], and Ang-2 expression in blood vessels was significantly correlated with the number of leucocytes in PDR, suggesting its capacity to modulate proinflammatory activities [32].

Although Ang-2 and VEGF signaling can act synergistically to promote vascular instability and neovascularization [16, 19, 20], Ang-2 may also act independently of VEGF to mediate vascular instability [19, 33]. In mouse models, Ang-2 overexpression was sufficient to mediate vascular leakage [34, 35], while Ang-2 inhibition alone stabilized retinal vessels, even under hypoxic or pericyte-deficient conditions [22]. Therefore, dual Ang-2/VEGF targeting may provide greater disease control (management of multiple pathways, allowing for the comprehensive management of factors including leakage, inflammation, and retinal fibrotic proliferation associated with the disease [3]) than anti-VEGF alone [24].

## Clinical lessons and insights from trials

Six Phase III randomized clinical trials (RCTs) [36–38] have compared faricimab (6 mg), a dual Ang-2/VEGF-A bispecific antibody, with aflibercept (unless otherwise noted, all subsequent references to aflibercept are related to the 2 mg dose), a VEGF and placental growth factor inhibitor [39]. The YOSEMITE/RHINE trials evaluated faricimab every 8 weeks (Q8W), or with personalized treat & extend (T&E) regimens up to every 16 weeks (Q16W), vs. aflibercept Q8W in patients with DME [36], while the TENAYA/LUCERNE trials evaluated faricimab given up to Q16W vs. aflibercept Q8W in patients with nAMD [37]; the duration of the trials was 2 years [40, 41]. The BALATON/COMINO trials of patients with ME due to RVO assessed monthly injections of faricimab vs. aflibercept 2 mg (up to Week 24; with all patients receiving faricimab thereafter, up to Week 72) [38]. In this paper, we translate the preclinical evidence regarding dual Ang-2/VEGF-A pathway inhibition to clinical biomarkers from large RCTs in order to better understand the improved disease control in nAMD, DME, and RVO.

## Dual Ang-2/VEGF-A inhibition: clinical biomarkers in retinal diseases

**Fig. 2:**
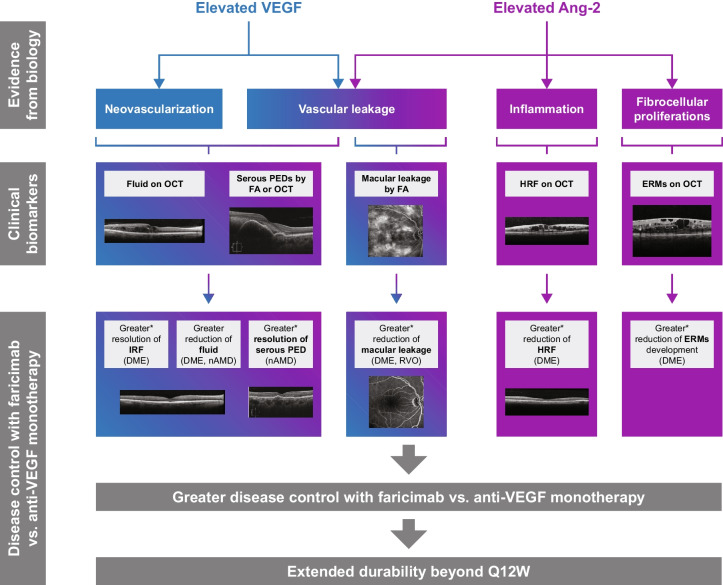
Role of VEGF and Ang-2 in the pathogenesis of retinal diseases and control of clinical biomarkers with dual VEGF/Ang-2 targeting [36, 42–49]. DME: YOSEMITE/RHINE trials; nAMD: TENAYA/LUCERNE trials; RVO: BALATON/COMINO trials. * Post hoc analyses not adjusted for multiplicity; no formal statistical conclusion should be made based on nominal p values. Ang-2, angiopoietin-2; DME, diabetic macular oedema; ERM, epiretinal membrane; FA, fluorescein angiography; HRF, hyperreflective foci; IRF, intraretinal fluid; nAMD, neovascular age-related macular degeneration; OCT, optical coherence tomography; PED, pigment epithelial detachment; Q12W, every 12 weeks; RVO, retinal vein occlusion; VEGF-A, vascular endothelial growth factor-A

Clinical biomarkers of vascular instability are important for establishing disease control and subsequent treatment decisions (Fig. 2).

## Biomarkers of neovascularization and vascular leakage

Optical coherence tomography (OCT) is used to measure/evaluate intraretinal fluid (IRF), subretinal fluid (SRF), PED fluid and central subfield thickness (CST) [50]. In DME, YOSEMITE/RHINE trial data showed that dual Ang-2/VEGF-A inhibition with faricimab Q8W/T&E resulted in greater mean CST reduction (secondary endpoint) compared with aflibercept after the head-to-head dosing period (Week 16; –169.9 μm /–174.5 μm vs. –151.7 μm), at 1 year (–206.6 μm/–196.5 μm vs. –170.3 μm), and this was maintained through 2 years (–209.4 μm/201.0 μm vs. –190.9 μm) [36, 42]. Similar results were observed in the Phase II RUBY trial in DME, which showed greater CST reduction with the combination of nesvacumab (Ang-2 inhibitor) and aflibercept vs. aflibercept alone [51]. A post hoc analysis of YOSEMITE/RHINE showed that faricimab achieved median time to first absence of IRF and all fluid (IRF and SRF) more than 9 months faster and with fewer injections vs. aflibercept [43]. In nAMD, post hoc analyses of TENAYA/LUCERNE showed that treatment with faricimab resulted in greater CST reductions vs. aflibercept after the head-to-head dosing period (Week 12; –145.4 μm vs. –133.0 μm) [44]. Furthermore, patients treated with faricimab experienced faster absence of IRF and SRF (by 4 weeks in the median time) with fewer injections, compared with aflibercept [42, 44]. Faricimab also reduced the presence (4% of patients with serous PEDs at baseline still had serous PEDs, vs. 12% with aflibercept) and thickness (PED mean thickness was 27.9 μm thinner with faricimab vs. aflibercept) of serous PEDs to a greater extent than aflibercept at Week 12 [45, 46]. Retinal pigment epithelium tears were associated with larger baseline PED height in TENAYA/LUCERNE; incidence (2–3%) was similar between treatment arms [45, 46] and comparable to findings from other trials of anti-VEGF-A monotherapy (2–3%) [52].

Reduction in macular leakage area on FA is an important biomarker that has been correlated with improved anatomical (reduced IRF volume and microaneurysm count) and best corrected visual acuity outcomes in patients with DME [53]. In YOSEMITE/RHINE, a post hoc analysis showed resolution of angiographic macular leakage in almost twice as many patients with faricimab vs. aflibercept (28% vs. 15%) after the head-to-head dosing period [47]. In BALATON/COMINO, a prespecified exploratory analysis showed that more patients achieved absence of angiographic macular leakage with faricimab than aflibercept (34%/44% vs. 21%/30%) at Week 24 (after the head-to-head dosing period) [47].

## Biomarker of inflammation

Hyperreflective foci (HRF) are detected with OCT and are defined as discrete, well-circumscribed, dot-shaped lesions up to 50 µm in diameter with equal or higher reflectivity compared with the retinal pigment epithelial band [54]. HRF are believed to be representative of activated microglia and may be a biomarker of an inflammatory microenvironment in retinal diseases [48, 54]; HRF reduction may be consistent with suppression of inflammatory pathways [48]. In a post hoc analysis of YOSEMITE/RHINE, in both the inner and outer retina, faricimab (Q8W and T&E regimens) reduced HRF number and volume more than aflibercept at Year 1 (p < 0.05 in all comparisons of faricimab vs. aflibercept) [48, 55].

## Biomarker of fibrotic proliferation

ERMs, identified using OCT, are a marker of retinal fibrotic proliferation [56] and have been observed in patients with DME [30]. In YOSEMITE/RHINE, presence of ERMs was assessed in a masked fashion by a reading center and defined using OCT as the presence of a membrane overlying the internal limiting membrane, causing significant macular architecture distortion in the central subfield [49]. In patients with no ERMs at baseline, per study entry criteria, faricimab Q8W treatment showed a greater reduction in the risk of development of ERMs over 2 years by more than 50% compared with aflibercept Q8W (proportion of patients with ERMs at Week 100: 4% vs. 8%) [49]. However, longer follow-up and additional studies are needed to determine the clinical implications of ERM development.

## Summary and clinical implications

Together, the data from six Phase III trials of the three disease states suggest that dual therapy Ang-2/VEGF-A inhibition with faricimab provides greater disease control than aflibercept 2 mg, by controlling neovascularization and vascular leakage (resolution of retinal anatomy, PEDs, and macular leakage; DME, nAMD, RVO) as well as by targeting inflammation (reduction of HRF; DME) and retinal fibrotic proliferation (reducing the risk of ERMs; DME) [42, 47–49].

We hypothesize that the observed biomarker benefits associated with faricimab compared with aflibercept 2 mg are likely attributable in part to the Ang-2 inhibition rather than solely the additional VEGF inhibition provided by faricimab’s higher molar anti-VEGF binding capacity. Indeed, numerous trials have explored higher molar doses of approved anti-VEGF therapies, revealing no or limited further reduction of CST with higher concentrations. For instance, in the HARBOR and READ-3 studies, ranibizumab doses of 2 mg and 0.5 mg achieved comparable CST reductions [57, 58]. In the PULSAR and PHOTON studies, aflibercept 8 mg achieved similar CST reductions as aflibercept 2 mg [59, 60]. Notably, the PHOTON study also demonstrated similar effects on macular leakage between aflibercept 8 mg and 2 mg during the head-to-head dosing period [61].

Conversely, faricimab reduced CST to a greater extent than aflibercept 2 mg in DME and nAMD [36, 42, 44], and also reduced macular leakage vs. aflibercept 2 mg in both DME and RVO [47]. Findings from the RUBY trial in DME conducted by Regeneron also support the role of Ang-2 in the anatomical response to faricimab. This trial revealed a greater reduction in CST with the addition of nesvacumab (an Ang-2 inhibitor) alongside aflibercept compared to aflibercept 2 mg alone [51], indicating potential additional benefits with dual Ang-2/VEGF inhibition. However, cross-trial comparisons between these higher-dose anti-VEGF trials and those involving faricimab must be interpreted with caution due to the differences in patient populations and study designs. Furthermore, as with any treatment, the risks with intravitreal injections of these agents – including potential endophthalmitis and intraocular inflammation – must be carefully considered alongside the clinical benefits associated with the control of the disease.

The biomarker evidence for faricimab, as outlined in the current manuscript, underscores the potential of faricimab to achieve greater control over neovascularization, vascular leakage, inflammation, and preretinal proliferation compared with aflibercept across various disease states. We hypothesize that these clinical benefits are a result of faricimab targeting two distinct pathways via Ang-2 and VEGF-A. The potential clinical significance for this differential biomarker effect, as it pertains to important patient outcomes, will need further exploration in future trials and real-world studies.

Up to 40% of patients with DME treated with anti-VEGF agents have persistent IRF and SRF accumulation after 3 years [23]. While this observation may be partly due to undertreatment, we hypothesize that pathways beyond VEGF are contributing to the exudation to some extent. Treatments aimed at achieving improved disease control with faster and more effective fluid resolution are important for improving patient outcomes. This includes improving patients' quality of life by preserving vision and potentially reducing clinic visits through increased treatment durability. Moreover, such advancements may alleviate treatment-related stress, minimize the use of transportation resources, decrease work absenteeism, and contribute to reducing carbon footprints [62]. Longer dosing intervals have been investigated in T&E regimens, which involve an initial monthly loading phase to maximize disease control and minimize anatomic signs of disease. Injection intervals are then modified based on the presence or absence of disease activity biomarkers such as retinal fluid, hemorrhage, or loss of vision [63–65]. In YOSEMITE/RHINE and TENAYA/LUCERNE, greater disease control with faricimab may have allowed for extended durability beyond every 12 weeks (Q12W). For example, in these trials, ~ 80% of patients with DME or nAMD achieved ≥ Q12W dosing at Year 2 with faricimab (Q16W, 62% and 63%, respectively) [44, 66]. Furthermore, over half of patients (56%) met the criteria for potential Q20W dosing intervals [67, 68].

In summary, modulation of both the Ang-2 and VEGF-A pathways with faricimab may provide greater disease control than with anti-VEGF monotherapy [69], leading to the potential for extended durability of Q12W or longer. Biomarker analyses revealed that faricimab not only leads to improvements in resolution of retinal fluid and macular leakage vs. aflibercept 2 mg [42, 44, 47], but also reduces HRF and risk of ERMs [48, 49]. Further research will continue to elucidate the importance of these clinical imaging biomarkers for evaluating retinal disease pathology/activity, their relationship with proteomic markers, and the impact of different treatment paradigms (fixed intervals/pro re nata/T&E) on disease control. Based on the evidence presented regarding disease control with a dual Ang-2/VEGF-A inhibitor, we hypothesize that earlier treatment with a dual pathway inhibitor has the potential to improve long-term patient outcomes; future evidence generation in terms of robust clinical trials is required to quantify the potential clinical benefit.
